# Advancing Primary Ciliary Dyskinesia Diagnosis through High-Speed Video Microscopy Analysis

**DOI:** 10.3390/cells13070567

**Published:** 2024-03-24

**Authors:** Wilfredo De Jesús-Rojas, Zachary J. Demetriou, José Muñiz-Hernández, Gabriel Rosario-Ortiz, Frances M. Quiñones, Marcos J. Ramos-Benitez, Ricardo A. Mosquera

**Affiliations:** 1 Department of Pediatrics and Basic Science, Ponce Health Sciences University, Ponce, PR 00716, USA; zdemetriou22@stu.psm.edu (Z.J.D.); grosario@psm.edu (G.R.-O.); frquinones@psm.edu (F.M.Q.);; 2 Department of Medicine, San Juan Bautista School of Medicine, Caguas, PR 00725, USA; josemh@sanjuanbautista.edu; 3 Department of Pediatrics, McGovern Medical School, University of Texas Health Science Center at Houston, Houston, TX 77030, USA; ricardo.a.mosquera@uth.tmc.edu

**Keywords:** primary ciliary dyskinesia, high-speed video microscopy analysis, Puerto Rico, ciliary dysfunction, diagnostic technology

## Abstract

Primary ciliary dyskinesia (PCD) is an inherited disorder that impairs motile cilia, essential for respiratory health, with a reported prevalence of 1 in 16,309 within Hispanic populations. Despite 70% of Puerto Rican patients having the *RSPH4A* [c.921+3_921+6del (intronic)] founder mutation, the characterization of the ciliary dysfunction remains unidentified due to the unavailability of advanced diagnostic modalities like High-Speed Video Microscopy Analysis (HSVA). Our study implemented HSVA for the first time on the island as a tool to better diagnose and characterize the *RSPH4A* [c.921+3_921+6del (intronic)] founder mutation in Puerto Rican patients. By applying HSVA, we analyzed the ciliary beat frequency (CBF) and pattern (CBP) in native Puerto Rican patients with PCD. Our results showed decreased CBF and a rotational CBP linked to the *RSPH4A* founder mutation in Puerto Ricans, presenting a novel diagnostic marker that could be implemented as an axillary test into the PCD diagnosis algorithm in Puerto Rico. The integration of HSVA technology in Puerto Rico substantially enhances the PCD evaluation and diagnosis framework, facilitating prompt detection and early intervention for improved disease management. This initiative, demonstrating the potential of HSVA as an adjunctive test within the PCD diagnostic algorithm, could serve as a blueprint for analogous developments throughout Latin America.

## 1. Introduction

The accurate diagnosis of primary ciliary dyskinesia (PCD), a genetic disorder characterized by dysfunctional motile cilia [1], is crucial for the timely treatment and management of the disease [2]. Motile cilia are essential for the proper functioning of various organ systems, and their impairment in PCD leads to a spectrum of clinical manifestations, notably chronic respiratory tract infections [1]. Globally, the disease is underdiagnosed, with a reported prevalence of approximately 1 in 16,309 within Hispanic populations [3], highlighting the need for improved diagnostic methodologies [3,4]. The field of PCD research is marked by significant controversies [5], particularly regarding the diagnostic criteria and the heterogeneity of genetic mutations associated with the disease [6,7]. While nasal nitric oxide (nNO) levels have been a traditional diagnostic marker [8], recent studies suggest that high-speed video microscopy analysis (HSVA) provides additional information about the ciliary function [9,10]. 

In Puerto Rico, the prevalence and specific microscopic characteristics of the cilia dynamics with the founder PCD mutation *RSPH4A* [c.921+3_921+6del (intronic)] are not defined [6], partly due to the limited access to diagnostic tools, including HSVA. This technology offers a detailed visualization of ciliary motion, enabling a deeper understanding of ciliary dynamics at a microstructural level [11]. To date, its application in the diagnosis algorithm of PCD in Puerto Rico has not been explored.

This study addresses this gap by implementing a protocol for HSVA for the first time in Puerto Rico to investigate PCD in a Puerto Rican cohort with the *RSPH4A* [c.921+3_921+6del (intronic)] founder mutation. By describing HSVA findings for this specific genetic mutation, which accounts for over 70% of PCD cases on the island, this work aims to significantly enhance the diagnostic accuracy for PCD in Puerto Rico. Moreover, our research contributes to the broader understanding of PCD as a global effort to understand this disease in developing countries in Latin America. 

## 2. Materials and Methods

### 2.1. Subjects

The study included a well-characterized cohort of patients with PCD (*n* = 12) with confirmed bi-allelic pathogenic *RSPH4A* [c.921+3_921+6del (intronic)] founder mutation and decreased nasal nitric oxide (nNO) levels, measured by a validated chemiluminescence technique (CLD 88sp Chemiluminescence Nitric Oxide Analyzer, Dürnten, Switzerland) as per protocol [3]. Only one patient was classified as a compound heterozygous patient, with two genetic variants in the *RSPH4A* gene [c.921+3_921+6del (intronic)] and c.1103T>G (p.Val368Gly)]. Nasal ciliated epithelial samples from all subjects with PCD were collected at their baseline health status, without a clinical history of viral or bacterial infection for over two weeks. A control group of 12 healthy subjects was recruited and compared with the patient cohort for statistical comparison.

### 2.2. Sample Collection and Preparation

Nasal ciliated epithelial samples were obtained via cytology brushing (Puritan Sterile Cytology Brushes). The nasal cytology brush was briefly inserted into one nostril and gently advanced to the inferior nasal turbinate with slight movements while performing an internal rotation of the brush and slightly pressing on the lateral wall of the inferior turbinate [12]. Once brushing was completed, immediate sample analysis was conducted. The nasal cytology brush sample was immersed in 3 mL of PneumaCult™-Ex Plus Medium to release any tissue that remained adherent to the bristles. The sample needed to be placed in sufficient media volume, allowing the full immersion of the brush. Samples were washed in Dulbecco’s Phosphate Buffered Saline (D-PBS), without calcium or magnesium, to remove mucus and debris, and centrifuged at 400× *g* for seven minutes to pellet the airway epithelial cells. After resuspension in 500 μL of PneumaCult™-Ex Plus Medium, the samples were incubated at 37 °C for 30 min before HSVA at 500 frames per second (fps), under a 40× and 60× lens magnification. 

### 2.3. Highspeed Video Microscopy Analysis (HSVA)

The sample analysis was conducted using the Nikon Eclipse Ti2 inverted microscope with a long working distance 40× and a 60× objective lens. An AOS PROMON U750 mono-chrome high-speed camera was attached to the microscope to capture high-speed video recording data. The camera had a light sensitivity grade of ISO 3600 and a sensor size of 4.8 μm pixels. This setup recorded samples at a frame rate of 500 fps. The AOS Imaging Software Version 4 was used to process the footage, which was uploaded via a USB3 cable. A microscope air table was employed in conjunction with the HSVA to minimize movement during the recording. The optimal resolution setting for HSVA was recorded at 500 fps, as recommended by the AOS Imaging software Version 4 in the camera suite and set at 880 × 637 pixels. Following the European Respiratory Society (ERS) guidelines [13], 2200 frames were recorded for five seconds at the specified resolution and under 40× and 60×. The recorded HSVA footage was saved as unprocessed image data (RAW) files. During the analysis, the region of interest (ROI) was not selected if there was mucus or debris on the surface interface of the cell cluster. To ensure representative footage of the ciliary population, an ROI was only selected if 10–15 equally distant adjacent ciliated cells were present. A microscope temperature control system (Life Imaging Services, Efringerstrasse 79, CH-4057 Basel, Switzerland) adapted to a Nikon Eclipse Ti2 Inverted microscope was used to maintain the extended observation of motile cilia at 37 °C to minimize variability in CBF. An intact cell lining was chosen over single cells to obtain a more representative sample [14]. The HSVA process was completed in less than 30 minutes per sample at 37 °C.

### 2.4. Manual Method for CBF Count

The CBF was manually calculated based on 10 complete beat cycles. This manual counting method adhered to protocols established in the literature, using the formula: Manual CBF (Hz) = (fps)/number of frames elapsed for 10 full ciliary beats) * 10. This formula facilitates the conversion to a per-beat-cycle basis [15,16]. To ensure precision in manual counting, each video was paused at the moment when the cilia approached their maximal bend. The video was advanced frame by frame until the cilia became fully bent, marking this as the starting frame. A tally was made of the total number of complete ciliary beats—encompassing both the effective and recovery strokes—from this starting point to the cycle’s final frame. The exact number of frames elapsed during 10 complete ciliary beats was carefully recorded for analysis.

### 2.5. Software Analysis

The Tracker Video Analysis (v.6.1.5) and Modeling tool, following Brown (2023), was used for CBP analysis [15]. Each video was captured at a high frame rate of 500 fps and subsequently uploaded into the Tracker program. We conducted a manual tracking process, focusing on the top-view videos to assess CBP. This involved a frame-by-frame review to identify the point of maximal ciliary bend. The ciliary tip location was marked and tracked across every five frames for 10 complete ciliary beat cycles. A graphical representation of the tracked ciliary tip was generated automatically, plotting oscillations in the x and y axes for the analysis.

### 2.6. Statistical Analysis

All statistical analyses were performed using the statistical software package GraphPad Prism version 10.1.1 for MacOS, developed by GraphPad Software, San Diego, CA, USA [www.graphpad.com (accessed on 18 January 2024)]. Descriptive statistics, including median and interquartile ranges (IQRs), were calculated to summarize the data, providing central tendency and dispersion measures. Mann–Whitney U tests were utilized for comparative analysis between unpaired groups possessing non-parametric continuous variables. P values of less than 0.05 were considered indicative of statistical significance.

## 3. Results

### 3.1. Subjects’ Characteristics and Demographics

In the study, 12 participants were recruited and underwent HSVA according to the study protocol [16]. The demographic details and clinical characteristics of PCD among the cohort are detailed in Table 1.

The participant cohort comprised a slight majority of females, with 7 out of 12 subjects (58%) being female. The median age of the subjects was 21 (IQR 14–45.5) years. Overall, 92% of the subjects exhibited homozygosity for the founder PCD mutation *RSPH4A* [c.921+3_921+6del (intronic)], while only one was compound heterozygous for the same gene *RSPH4A* [c.921+3_921+6del (intronic)] plus *RSPH4A* c.1103T>G (p.Val368Gly). All participants (12/12, 100%) were found to have bronchiectasis on a chest computed tomography (CT) scan. None of the patients displayed laterality defects, accounting for 0/12, 0% of the cohort. Chronic cough, defined as a persistent, year-round wet cough that begins before six months of age, was present in all subjects (12/12, 100%). Neonatal respiratory distress and chronic otitis media were prevalent in half of the patients, with each condition affecting 6/12, 50% of the cohort. Chronic sinusitis was a universal finding in 12/12, 100% of the patients. Hearing loss was identified in 7/12, 58% of the subjects. A notable 10/12, 83% of the cohort showed colonization with Pseudomonas species, and (1/12, 8%) had a history of Burkholderia cepacia infection. All patients in the study exhibited nasal Nitric Oxide (nNO) levels below the diagnostic threshold of 77 nL/min. Only one patient was a recipient of a double lung transplant. 

### 3.2. Ciliary Beat Frequency (CBF) Measurement and Ciliary Beat Pattern (CBP) Assessment

**Figure 1:**
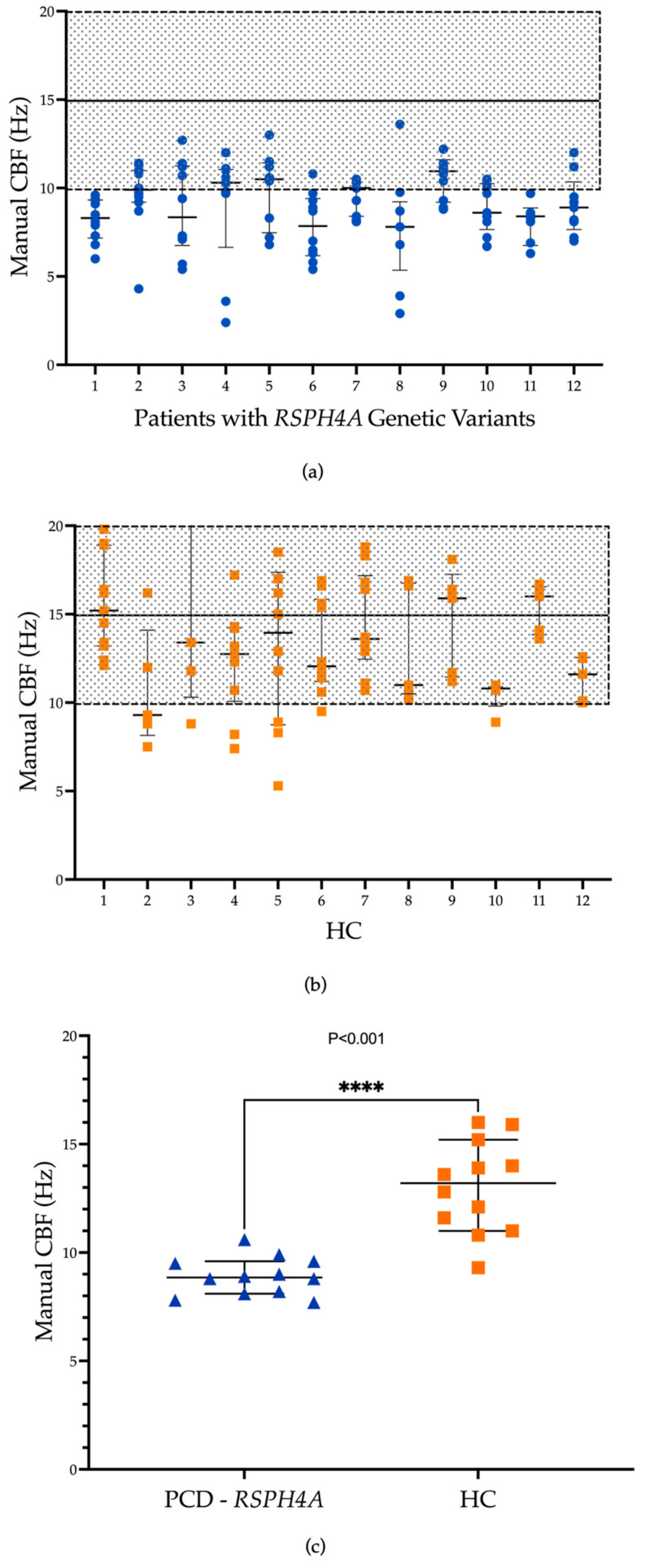
Ciliary beat frequency (CBF) measurement in patients with PCD with the *RSPH4A* [c.921+3_921+6del (intronic)] founder mutation (**a**) and healthy controls (**b**). This figure illustrates the CBF obtained from a series of samples measured using the manual method. The y-axis represents the CBF in Hertz (Hz), and the x-axis enumerates the individual patients with median measurements across each sample set. (**c**) Comparison of median CBF (Hz) between patients with PCD with the founder mutation versus healthy controls. Mann–Whitney test between cohorts showed statistical significance (*p* < 0.001, ****) among Median CBF. The graph’s dotted area and solid line denote the normal CBF range and median typically observed in healthy individuals, as in previous publications [17]. Patient with PCD #12 has a compound heterozygous for the *RSPH4A* [c.921+3_921+6del (intronic)] plus *RSPH4A* c.1103T>G (p.Val368Gly).

**Figure 2:**
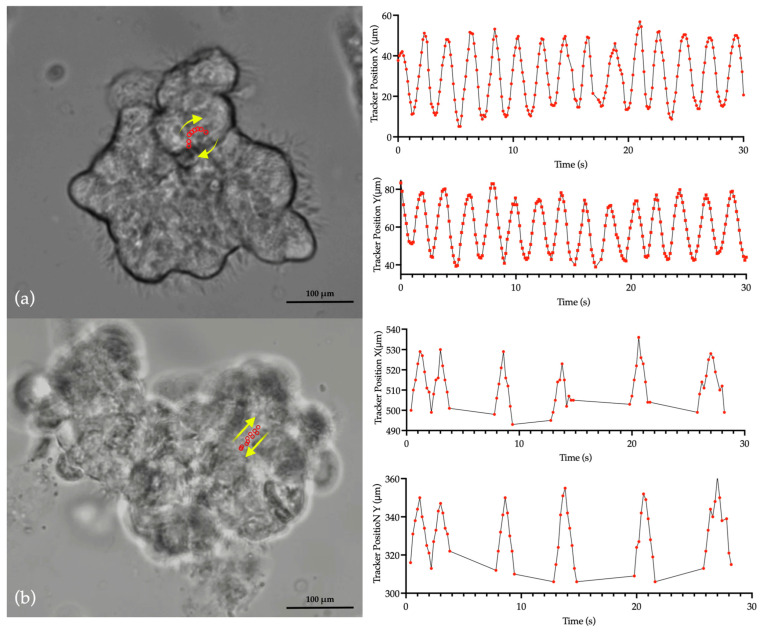
Representative illustration of nasal ciliary biopsy dynamics and ciliary beat pattern (CBP). Panel (**a**) presents a top view of the nasal ciliated epithelium from a patient homozygous for the *RSPH4A* [c.921+3_921+6del (intronic)] founder mutation, illustrating the cilia’s rotational motion. As quantified by tracker software, this pattern is characterized by wide-ranging oscillations at the cilia tips along the x and y axes. Panel (**b**) displays a top view from a healthy control subject, where cilia demonstrate the expected bidirectional movement, with pronounced and distinct oscillations observable on the x and y axes, also captured and analyzed with tracker software. An accompanying video that provides a dynamic visualization of the *RSPH4A* [c.921+3_921+6del (intronic)] founder mutation patterns (Video S1) as compared with healthy control (Video S2) is available in the Supplemental Material.

Figure 1a,b present CBF measurements obtained from a sample set using a manual method in patients with PCD due to *RSPH4A* genetic variants and healthy controls. The manual method yielded a CBF median of 9.3 Hz that ranged from 7.9 to 10.9 Hz for patients with PCD and 13.1 with a range of 9.3 to 16 in the healthy control group. The Mann–Whitney test revealed a statistically significant difference in median CBF (Hz) between patients with PCD carrying the founder mutation versus healthy controls (*p* < 0.001, ****), Figure 1c. CBP, as analyzed using tracker software (v.6.1.5), exhibited a rotational motion in all samples (100%) when observed in top-view videos captured by HSVA as compared with a bidirectional movement on healthy control samples; see Figure 2.

## 4. Discussion

This study represents a pioneering effort to advance the diagnosis of PCD in Puerto Rico by implementing, for the first time, the HSVA technique in Puerto Ricans with a PCD founder mutation. Daniels et al., 2013, represents the only study that has assessed CBF and CBP using HSVA at room temperature in a cohort of six patients of Puerto Rican Hispanic descent in the United States [18]. While the average CBF reported in their study was slightly lower than we observed, both studies identified CBF values below the established normal threshold for healthy individuals [17]. In contrast, our study analyzed each sample at 37 °C to avoid temperature effects [19]. Furthermore, their findings on CBP are consistent with ours, demonstrating a rotational movement in the top-view analysis of the cilia. The *RSPH4A* gene, which was the focus of our study, encodes a protein integral to the central apparatus and is implicated in the 9 + 2 microtubular organization [20]. Defects in this central complex are known to be linked to atypical waveforms similar to those observed in the 9 + 0 nodal cilia, which exhibit a circular motion [10].

In Europe, the introduction of HSVA has proven highly beneficial by providing a much-needed enhancement to the evaluation process, revealing critical insights into the ciliary dysfunction associated with several PCD-related genetic variants [9]. By evaluating this technique in patients with the *RSPH4A* [c.921+3_921+6del (intronic)] founder mutation, we were able to characterize the low CBF coupled with the rotational CBP, which aligns with the mutation’s expected physiological impact and PCD clinical outcomes similar to other described genetic variants in *RSPH4A* and *RSPH9* genes [21]. The consistency of the results obtained reinforces the credibility of HSVA as a complementary diagnostic tool for PCD associated with the *RSPH4A* founder mutation. The utility of HSVA lies in its capacity for the ex vivo assessment of ciliary motion, which provides an objective analysis of CBF and CBP. Moreover, HSVA minimizes the potential for human error typically associated with the assessment of ciliary function using conventional microscopy, thus providing valuable corroborative data to support clinical diagnoses of PCD. This is particularly beneficial in clinical environments where expedited patient evaluation is imperative, genetic testing may involve longer processing times, or other diagnostic tools are unavailable. 

Furthermore, aligning results obtained from well-established and standardized protocols to perform HSVA attests to its accuracy, providing a complementary tool to the algorithm of PCD diagnosis. Ensuring consistency in applying HSVA across various PCD centers is essential when adopting new technologies within clinical practices. Such standardization is key to maintaining and enhancing the quality of patient care and minimizing the risk of misdiagnosing PCD as we advance diagnostic techniques. By adopting HSVA, Puerto Rico elevates its local healthcare standards and joins the international PCD community in adopting cutting-edge techniques for PCD diagnosis. This initiative is committed to improving patient outcomes and contributes to the global endeavor to understand and treat PCD more effectively. Moreover, the presence of ciliary dysfunction with reduced nNO levels below the established diagnostic threshold further substantiates the clinical utility of HSVA in the identification and characterization of PCD, as 100% of our patients had low nNO levels, low CBF, and rotational CBP on HSVA. 

Given the current diagnostic resources in Puerto Rico and following guidelines from the American Thoracic Society (ATS), ERS, and the PCD Foundation, we have developed a population-targeted diagnostic algorithm for PCD in Puerto Rico. The algorithm specifically addresses the high prevalence of the *RSPH4A* founder mutation within the Puerto Rican population and our available diagnostic modalities. This focused algorithm for Puerto Rico complements, rather than replaces, existing guidelines. The diagnostic process starts with a comprehensive clinical assessment aimed at detecting symptoms and physical indicators of PCD, when cystic fibrosis has been excluded. All patients should undergo evaluation at a center accredited by the PCD Foundation and able to perform nNO level screening using a validated chemiluminescence technique. The evaluation must include nNO levels as a non-invasive screening method during the initial visit and a follow-up measurement two weeks later, provided the patient’s respiratory symptoms are at baseline. Subsequent steps involve collecting a sample for HSVA to assess CBF and CBP, followed by an in-depth genetic panel test targeting the *RSPH4A* founder mutation to confirm PCD diagnosis.

The combination of nNO levels with low CBF and rotational CBP on HSVA, and genetic testing showing pathogenic variants on the *RSPH4A* gene, confirm the most common phenotype associated with PCD in Puerto Rico. 

**Figure 3:**
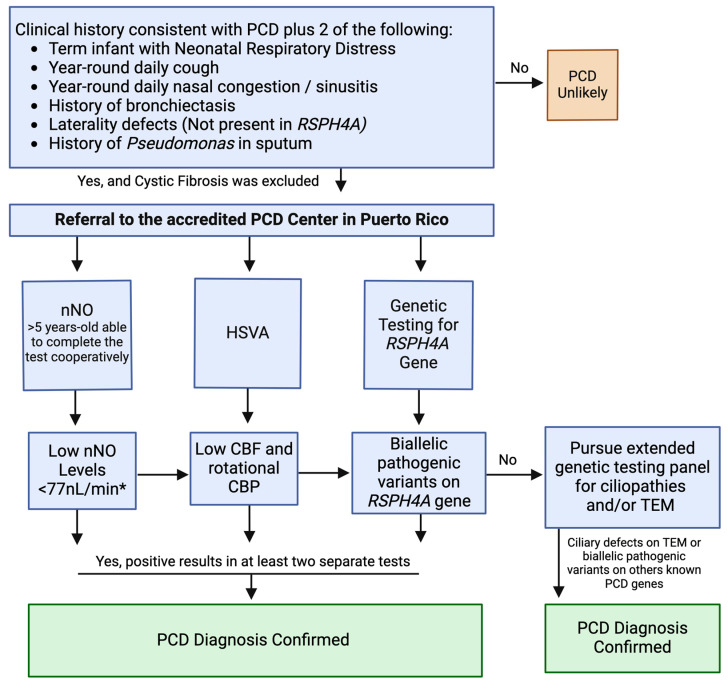
Population-targeted diagnostic algorithm for PCD in Puerto Rico. This algorithm presents a population-targeted diagnostic algorithm for PCD specifically designed for the Puerto Rican demographic, considering the region’s high prevalence of the *RSPH4A* founder mutation. (*): Threshold values below 77 nL/min are considered positive for PCD at baseline status in two separate visits.

If a patient is unable to complete the nNO, the HSVA results are inconclusive, or genetic testing is negative, a more comprehensive evaluation should be pursued, including repeat HSVA, a nasal biopsy for transmission electron microscopy (TEM), and/or broader genetic testing (whole exome sequencing) if resources allow the evaluation of other ciliopathies or immunodeficiencies. The algorithm promotes a multidisciplinary approach to management and encourages regular follow-up for ongoing patient care. It is vital to update the algorithm periodically, reflecting new scientific insights and the implementation of new diagnostic tools and ensuring it remains applicable to the healthcare context of Puerto Rico. The algorithm (Figure 3) not only serves the immediate need for PCD diagnosis but also contributes to the broader goal of improving rare disease management on the island. 

Our findings echo the existing body of research that underscores the heterogeneity of PCD presentations and the corresponding need for comprehensive diagnostic approaches [22]. Integrating HSVA as part of the evaluation for patients with PCD could significantly enhance our comprehension of the disease, especially for populations like Puerto Ricans that present with unique founder mutations exhibiting rotational CBP. This study highlights the pairing of genetics to clinical and functional observations to increase our understanding of PCD across different populations and to comprehensively address the challenges in securing a definitive diagnosis. As the field moves towards the implementation of advanced tools like HSVA to offer deeper insights into specific PCD phenotypes and ciliary behaviors, it raises the necessity of following rigorous protocols and ensuring standardization across diagnostic platforms. Future studies are needed to explore the utility of new open-source software such as Cilialyzer and CiliarMove to provide a standardized approach to measuring CBF [23,24]. Although manual counting has demonstrated a reasonable and easy methodology to measure CBF directly, proving reliable, objective, and accessible software tools may offer a standardized measurement system to facilitate the comparison and translatability of the findings across different cohorts.

While our study provides a significant leap forward in PCD diagnostics in Puerto Rico, there are limitations of the study to be considered. The sample size, though sufficient for a preliminary analysis in rare diseases, necessitates expansion in future studies to validate and generalize our findings. However, few studies have evaluated HSVA in cohorts with a specific and unique founder mutation [18]. Our study did not extend to assessing the CBF of ciliated epithelial cells within air–liquid interface (ALI) cultures [25]. This decision was strategically made, as our primary objective was to establish a rapid and cost-efficient diagnostic protocol that could be readily implemented in a clinical setting. While ALI cultures represent a more physiologically relevant model [26], they require significant time and resources to establish. Our approach, focusing on direct sampling methods, allowed for immediate analysis and offered a balance between clinical efficiency and CBF accuracy. Future studies incorporating ALI culture assessments are needed to complement and expand upon our findings, further enriching the understanding of ciliary function in PCD within the context of native epithelial architecture. We also acknowledge a limitation of our study regarding the genetic diversity of the patient cohort. While our diagnostic approach has effectively identified the characteristic decreased CBF and distinctive rotational CBP associated with the *RSPH4A* founder mutation, our analysis was not extended to PCD patients with genetic mutations other than *RSPH4A* variants. This is principally because our cohort predominantly consists of patients with a homogenous presentation of the founder mutation, reflecting the genetic landscape of the Puerto Rican population we studied. Future research would benefit from including a broader spectrum of genetic mutations to comprehensively evaluate the diagnostic capacity of HSVA across different PCD genotypes.

The successful application of HSVA for PCD diagnosis in Puerto Rico represents a significant stride in addressing the diagnostic challenges associated with this rare disease. Our work not only enhances the diagnostic capabilities on the island but also contributes to the global effort to understand and manage PCD in diverse populations, specifically those with founder mutations.

## 5. Conclusions

Our research has fundamentally advanced the diagnosis of PCD by integrating several diagnostic modalities in Puerto Rico. The HSVA was implemented for the first time in a Puerto Rican cohort in this study. This diagnostic approach has described the characteristic low CBF and distinctive rotational CBP associated with the *RSPH4A* founder mutation. Our approach emphasizes the importance of these diagnostic markers in evaluating PCD. The implications of our study are important, considering HSVA as a valuable tool that could reshape the identification, management, and research of this rare disease in Puerto Rico and throughout Latin America.

## Figures and Tables

**Table 1:** Patients with PCD demographics and diagnostic testing.

Characteristics:	Percentage (%):
Gender (F)	58
*RSPH4A* Homozygosity	92
*RSPH4A* Compound Heterozygous	8
Bronchiectasis	100
Laterality Defects	0
Chronic Cough *	100
Neonatal Respiratory Distress	50
Chronic Sinusitis	100
Chronic Otitis Media	50
Hearing loss	58
*Pseudomonas* Colonization	83
Infection with *Burkholderia Cepacia*	8
Lung Transplant Recipient	8
nNO Levels below 77 nL/min	100

F: female; * persistent, year-round wet cough that started before 6 months of age.

## Data Availability

The datasets generated and/or analyzed during the current study are not publicly available due to privacy restrictions but are available from the corresponding author upon reasonable request.

## Supplementary Materials

The following supporting information can be downloaded at: https://www.mdpi.com/article/10.3390/cells13070567/s1. Video S1: Dynamic visualization of the cilia’s rotational motion in the nasal ciliated epithelium from a patient homozygous for *RSPH4A* [c.921+3_921+6del (intronic)], with tracker software capturing the pattern characterized by extensive oscillations at the cilia tips; Video S2: Dynamic visualization of the typical bidirectional ciliary motion seen in the nasal ciliated epithelium from a healthy control subject.
